# Early Atherosclerotic Changes in Coronary Arteries are Associated with Endothelium Shear Stress Contraction/Expansion Variability

**DOI:** 10.1007/s10439-021-02829-5

**Published:** 2021-07-29

**Authors:** Valentina Mazzi, Giuseppe De Nisco, Ayla Hoogendoorn, Karol Calò, Claudio Chiastra, Diego Gallo, David A. Steinman, Jolanda J. Wentzel, Umberto Morbiducci

**Affiliations:** 1 PoliToBIOMed Lab, Department of Mechanical and Aerospace Engineering, Politecnico di Torino, Corso Duca degli Abruzzi, 24, 10129 Turin, Italy; 2 Department of Cardiology, Biomedical Engineering, Erasmus MC, 3000 CA Rotterdam, The Netherlands; 3 Biomedical Simulation Laboratory, Department of Mechanical & Industrial Engineering, University of Toronto, Toronto, Canada

**Keywords:** Atherosclerosis, Wall shear stress, Computational fluid dynamics, Topological skeleton, Plaque progression, Topological shear variation index

## Abstract

Although unphysiological wall shear stress (WSS) has become the consensus hemodynamic mechanism for coronary atherosclerosis, the complex biomechanical stimulus affecting atherosclerosis evolution is still undetermined. This has motivated the interest on the contraction/expansion action exerted by WSS on the endothelium, obtained through the WSS topological skeleton analysis. This study tests the ability of this WSS feature, alone or combined with WSS magnitude, to predict coronary wall thickness (WT) longitudinal changes. Nine coronary arteries of hypercholesterolemic minipigs underwent imaging with local WT measurement at three time points: baseline (T1), after 5.6 ± 0.9 (T2), and 7.6 ± 2.5 (T3) months. Individualized computational hemodynamic simulations were performed at T1 and T2. The variability of the WSS contraction/expansion action along the cardiac cycle was quantified using the WSS topological shear variation index (*TSVI*). Alone or combined, high *TSVI* and low WSS significantly co-localized with high WT at the same time points and were significant predictors of thickening at later time points. *TSVI* and WSS magnitude values in a physiological range appeared to play an atheroprotective role. Both the variability of the WSS contraction/expansion action and WSS magnitude, accounting for different hemodynamic effects on the endothelium, (1) are linked to WT changes and (2) concur to identify WSS features leading to coronary atherosclerosis.

**Supplementary Information:**

The online version contains supplementary material available at 10.1007/s10439-021-02829-5.

## Introduction

Among the biomechanical factors, local hemodynamics is a recognized promoter of the initiation and progression of atherosclerotic disease in coronary arteries.^4^,^36^,^47^ A large body of literature has supported the consistency of the ‘hemodynamic risk hypothesis’ in coronary disease, emphasizing the crucial and multifaceted role played by wall shear stress (WSS) in conditioning the initiation, localization, and growth of coronary lesions.^4^ In this scenario, an arsenal of hemodynamic descriptors has been proposed as markers of flow disturbances,^41^,^43^,^45^ aiming to quantify the impact of different WSS features in promoting focal endothelial dysfunction and inflammation^4^, and affecting local blood-to-wall mass transfer rates.^20^,^23^

Low/oscillatory WSS has become the consensus hemodynamic mechanism for coronary atherosclerosis.^6^,^26^,^28^,^36^,^43^,^47^ However, low/oscillatory WSS may describe only partially the biomechanical stimulus affecting coronary atherosclerosis evolution.^40^ Previous studies underlined that the presence of low WSS alone may not be sufficient to predict the locations of future coronary plaques.^41^ Pedrigi *et al*. (2015), by inducing regions of persistent disturbed blood flow within the coronary arteries of hypercholesterolemic minipigs through a perivascular cuff, demonstrated that, besides low WSS, complex WSS features including multidirectionality have a causal role in the initiation of coronary atherosclerotic lesions.^39^ Moreover, recent evidences attributed a role for multidirectional WSS in coronary plaque progression and changes in plaque composition.^21^,^25^ Finally, the emerged weak-to-moderate capability of low, oscillatory or multidirectional WSS to identify/predict coronary lesion localization and development may indicate a more complex mechanistic action of the shear forces on the endothelium.

In this regard, a marked interest has recently emerged on the topological skeleton analysis applied to the WSS vector field.^1^,^2^,^33^,^34^ The WSS vector field topological skeleton consists of fixed points, where the WSS vector vanishes, and manifolds, namely the regions connecting them, where the WSS vector field contracts or expands. The interest in such an analysis is dictated by the fact that: (1) it is instrumental in identifying flow features usually classified as disturbed flow, in the sense that they have been put in relation with “aggravating” biological events involved in atherosclerosis;^33^ (2) it can be used to quantify the contraction/expansion action exerted by the WSS on the endothelium. Very recent findings have suggested a link between the WSS topological skeleton and adverse vascular response. In detail, the variation along the cardiac cycle of the WSS contraction/expansion action at the luminal surface has emerged as an indicator of wall degradation in the ascending thoracic aorta aneurysm^12^ and as a predictor of long-term restenosis risk in the carotid bifurcation after endarterectomy.^37^

Based on those links,^12^,^37^ this study tests the ability of WSS topological skeleton features alone or in combination with WSS to predict the temporal evolution in coronary artery wall thickness (WT), a hallmark of atherosclerosis development, in an atherosclerotic pig longitudinal study.^10^,^11^,^21^ To do that, WSS topological skeleton features were obtained from individualized computational hemodynamics simulations, adopting a recently proposed Eulerian-based method,^37^ and compared to changes in WT over time, measured *in vivo*. The final aim is to probe whether fluid mechanic quantities giving a direct measure of the variability of the contraction/expansion action exerted by the blood flow on endothelial cells (ECs) are capable to account for longitudinal local WT changes.

## Materials and Methods

### Animal Population and Imaging

**Figure 1:**
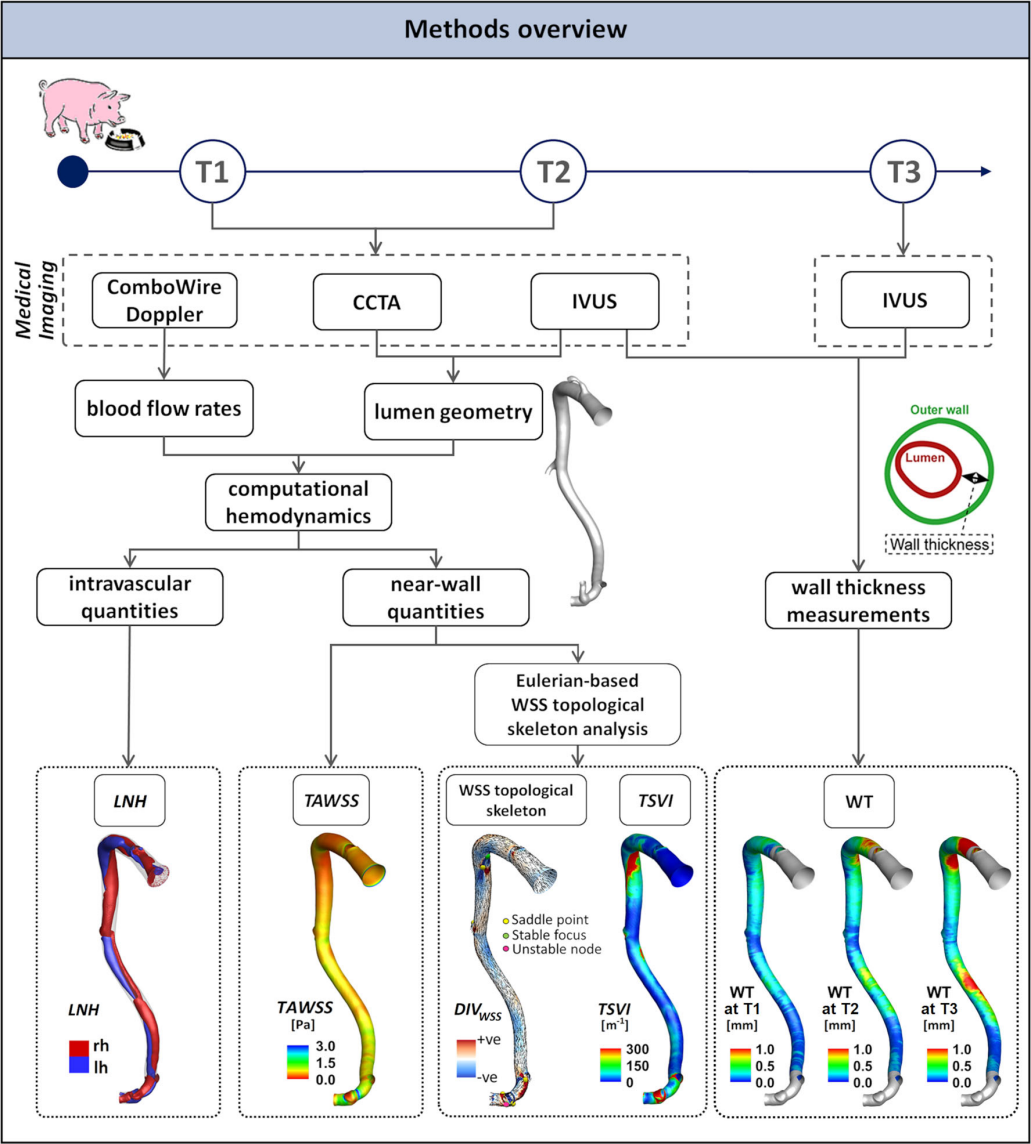
Schematic diagram of the study design, showing how imaging data contribute to define vessel geometry, hemodynamic variables, and wall thickness measurements. CCTA: coronary computed tomography angiography, IVUS: intravascular ultrasound, WT: wall thickness, WSS: wall shear stress, *LNH* local normalized helicity indicating right-handed (rh) and left-handed (lh) helical blood structures, *TAWSS* time-average wall shear stress, *TSVI* topological shear variation index, *DIV*_*WSS*_ divergence of the normalized WSS vector field.

An overview of the methods is provided in Fig. 1. Adult familial hypercholesterolemia Bretoncelles Meishan mini-pigs with a low-density lipoprotein receptor mutation were put on a high fat diet to trigger atherosclerosis development. Details of the animal study protocol are reported elsewhere.^18^,^28^ Of the original set of animals,^11^,^21^ only five exhibited a high atherosclerotic plaque progression rate. One pig died early due to myocardial infarction, and one other was sacrificed due to an acute occlusion of a femoral artery. The longitudinal data of the remaining animals (*N* = 3) were used for the study. For each animal, the three main coronary arteries (left anterior descending—LAD, left circumflex—LCX, and right coronary artery—RCA) were imaged by coronary computed tomography angiography (CCTA) and intravascular ultrasound (IVUS), resulting in a total of nine coronary models. CCTA and IVUS acquisitions were performed at 3 months (T1, considered as the baseline in this study), at an average of 8.6 months (T2), and at an average of 10.6 months (T3) after the start of the diet. Combowire Doppler (Volcano Corp., Rancho Cardova, CA, USA) flow velocity measurements were acquired in each artery at the inflow section and immediately upstream and downstream from each side branch, at T1 and T2, as reported in detail in previous studies.^10^,^11^,^21^

The study, performed according to the National Institute of Health guide for the Care and Use of Laboratory animals^8^, obtained the ethical approval from the local animal ethics committee of the Erasmus MC (EMC nr. 109-14-10).

### Wall Thickness Measurements

Quantitative analysis of WT was carried out using the QCU-CMS software (version 4.69, Leiden University Medical Centre, LKEB, Division of Image Processing). In detail, for each coronary vessel (3 LAD, 3 LCX and 3 RCA), maps of local WT were obtained on IVUS images at T1, T2, and T3 by using a semi-automatic method for the lumen and vessel wall contour detection.^10^,^21^ Technically, per Fig. 1, WT was locally measured by subtracting the distance value between lumen centroid and lumen contour (i.e., local lumen radius) to the distance value between lumen centroid and outer wall contour (i.e., local outer wall radius). WT measurements were averaged over 3 mm/45 degrees sectors of the luminal surface, to analyse their possible relation with hemodynamics, according to previous studies.^10^,^21^

### Computational Hemodynamics

The 3D geometry of each coronary artery (Figure S1 of the Supplementary Materials, where animals were named as pig A, B and C) was reconstructed at T1 and T2 (N = 18) by aligning the IVUS lumen contours along the centreline extracted from the corresponding 3D geometry reconstructed from CCTA. Details on 3D geometry reconstruction are extensively reported elsewhere.^11^,^13^ The reconstructed geometries were discretized with tetrahedral elements and 5 near-wall prismatic layers, resulting in a mesh cardinality of 6 million elements on average. Unsteady-state CFD simulations were performed to characterize coronary hemodynamics (Fig. 1). The governing equations of fluid motion were numerically solved by using the finite volume method. Blood was assumed as an incompressible, homogeneous, non-Newtonian fluid.^11^ In detail, blood was assumed to present the same rheological behaviour as a Carreau fluid. ComboWire Doppler velocity measurements were used to derive individualized boundary conditions according to the strategy detailed in the Supplementary Material. Descriptive notes on CFD settings, extensively detailed elsewhere,^10^,^21^ are briefly reported in the Supplementary Materials.

### WSS Topological Skeleton Analysis

**Figure 2:**
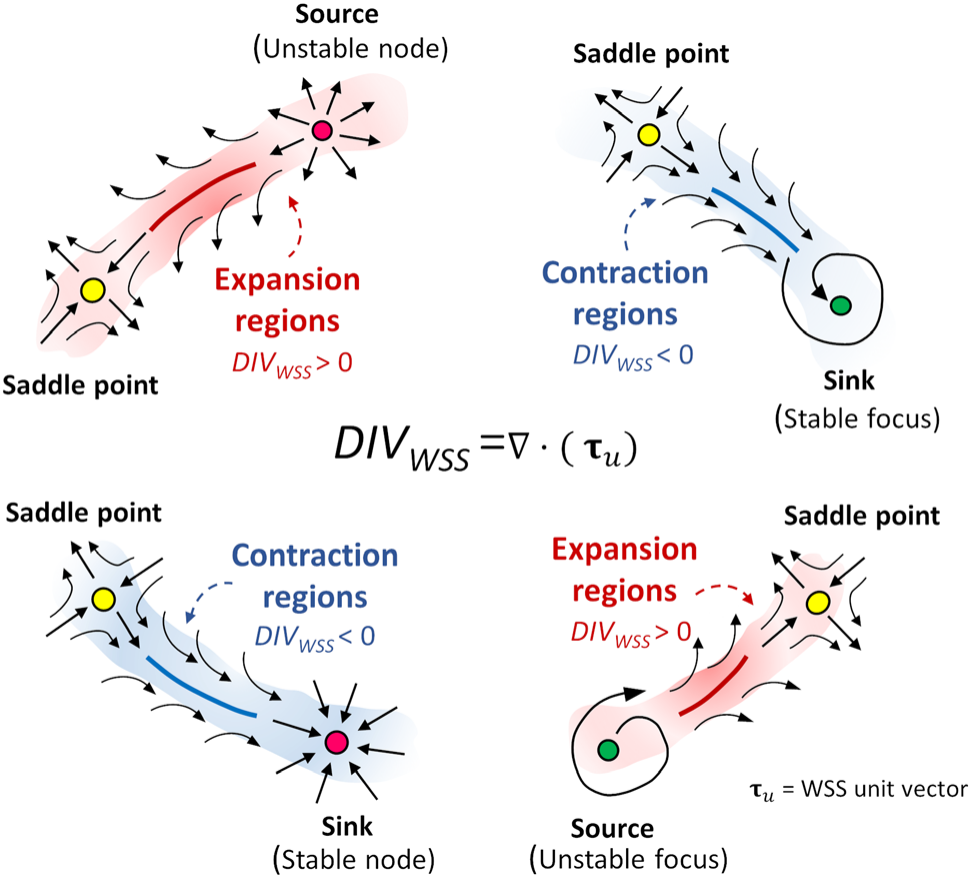
Explanatory sketch of topological features of the WSS vector field. Configuration of WSS contraction/expansion regions, colored by blue/red respectively, is presented together with their associated fixed points, which can be of different type (saddle points, nodes, foci) and nature (stable, unstable).

The WSS vector field at the luminal surface of each coronary model, obtained as an output of the CFD simulations, was analysed. In particular, the features of the WSS topological skeleton were investigated by applying a recently proposed Eulerian-based approach.^33^ The WSS topological skeleton consists of fixed points, i.e., points where WSS is equal to zero, and stable/unstable manifolds inherently linking fixed points. Stable/unstable manifolds identify patterns of the WSS vector field exerting an expansion/contraction action onto the luminal surface of the vessel.^12^,^33^,^34^,^37^ An explanatory sketch of possible configurations of WSS topological skeleton features is presented in Fig. 2.

As described elsewhere,^12^,^33^,^37^ the divergence of the normalized WSS vector field can be used to easily identify WSS manifolds:

1 $$DIV_{\text{WSS}} = \nabla \cdot \left( {\frac{{\user2{\tau }} }{{\parallel {\user2{\tau }} \parallel_{2} }}} \right) = \nabla \cdot\left( {{\user2{\tau }}_{\text{u}} } \right),$$

where ${\user2{\tau }}$ and ${\user2{\tau }}_{\text{u}}$ are the WSS vector and its unit vector, respectively (i.e., ${\user2{\tau }}_{\text{u}}$ is the WSS vector normalized by its magnitude, $\parallel {\user2{\tau }} \parallel_{2}$). More in detail, the WSS spatial contraction/expansion configuration patterns can be identified through Eq. (1), which quantifies the rate of change of directionality of nearby WSS trajectories by neglecting the vector field magnitude variation.^12^,^33^,^37^ In physical terms, negative values of *DIV*_WSS_ at the luminal surface of the vessel identify regions where WSS exerts a contraction action on the endothelium and approximates attracting manifolds, while positive values of *DIV*_WSS_ identify regions where WSS exerts an expansion action and approximates repelling manifolds (Fig. 2).

In the Appendix, the proof for the use of the normalized WSS divergence to identify WSS manifolds is detailed. The applicability of the recently proposed WSS divergence-based method for the analysis of the WSS topological skeleton, developed for Newtonian fluids,^33^ is extended here to the class of Reiner-Rivlin fluids called generalised Newtonian fluids, such as Carreau fluids.^42^

According to the scheme proposed in a previous study,^33^ the Poincaré index^18^ was considered for WSS fixed points identification. Then, the eigenvalues of the Jacobian (WSS derivative) matrix were used to classify the fixed point as: (1) a sink, if it attracts nearby trajectories; (2) a source, if it repels nearby trajectories; (3) saddle point, if it attracts and repels nearby trajectories along different directions. Typically, due to the inherent conservation properties of the Poincaré index, a contraction region starts from a saddle point and vanishes into a sink (stable node or stable focus, as depicted in Fig. 2). Contrarily, an expansion region starts from a source (unstable node or unstable focus) and vanishes into a saddle point (Fig. 2). The occurrence of instantaneous WSS fixed points (saddle points, sources, and sinks) at the coronary luminal surface was quantified to qualitatively investigate the instantaneous variability of WSS topological skeleton along the cardiac cycle and between time points T1 and T2 of the study.

In addition, based on the demonstrated physical meaning of Eq. (1), the variability of WSS contraction and expansion action exerted on the endothelium along the cardiac cycle was here quantified by adopting the quantity topological shear variation index (*TSVI*), defined as the root mean square deviation of the divergence of the normalized WSS with respect to its average over the cardiac cycle^12^,^37^:

2 $$TSVI = \left\{ {\frac{1}{T}\mathop \smallint \limits_{0}^{T} \left[ { \nabla \cdot\left( {{\user2{\tau }}_{\text{u}} } \right) - \overline{{\nabla \cdot({\user2{\tau }}_{\text{u}} )}} } \right]^{2} {dt}} \right\}^{1/2} ,$$

where *T* is the cardiac cycle period. Thus, *TSVI* is an integral measure of the local unsteady nature of the WSS manifolds along the cardiac cycle, and hence of the heterogeneous action of the hemodynamic forces to which ECs may be focally exposed.

To complement the analysis of the WSS topological skeleton, the luminal distribution of time-averaged wall shear stress (*TAWSS*) magnitude along the cardiac cycle was also evaluated, as the exposure to low *TAWSS* values is widely recognized as a promoter of atherosclerotic disease.^47^

**Figure 3:**
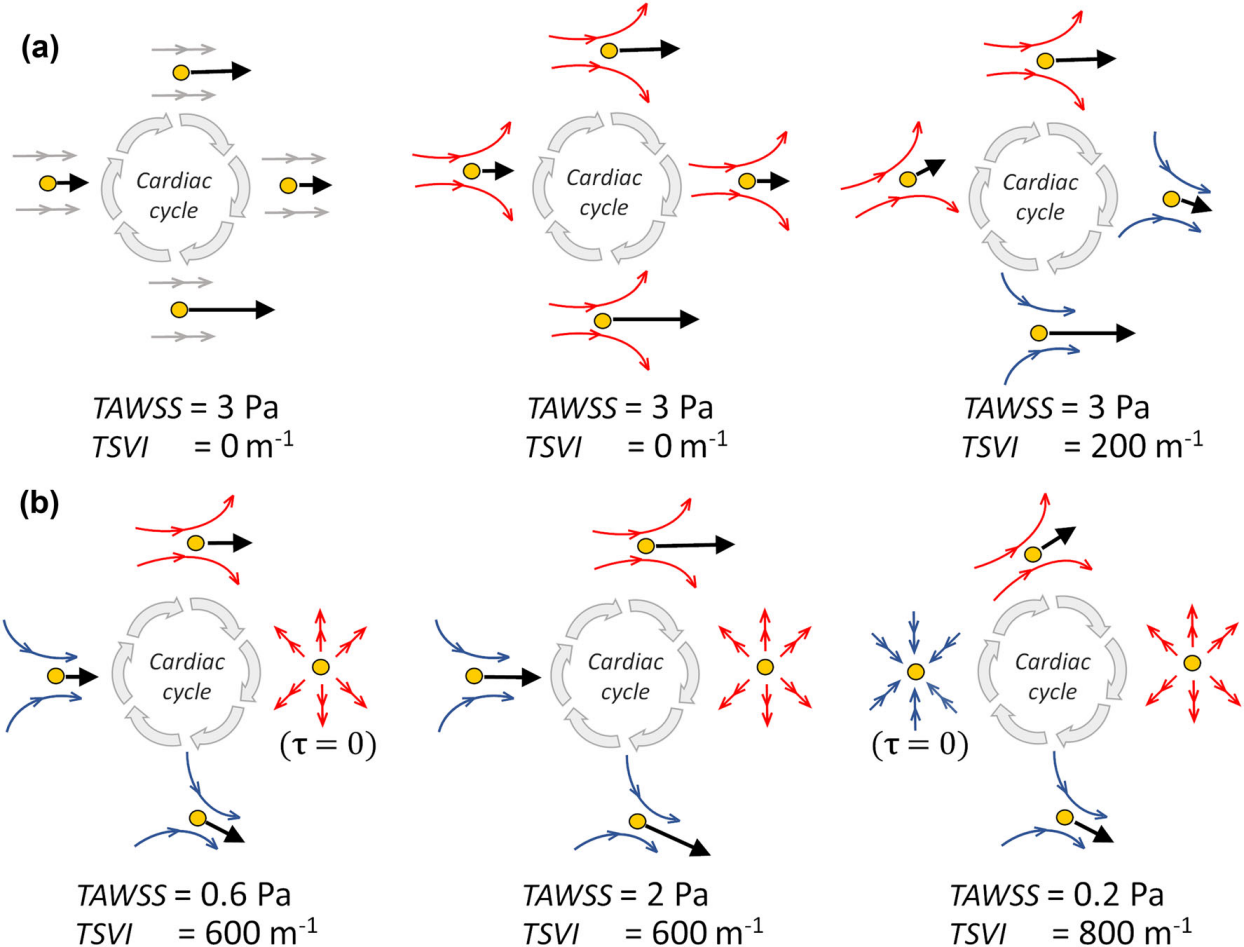
Six different flow environments to which an EC could be exposed. Black arrows represent instantaneous WSS vectors (${\varvec{\uptau}}$) applied to a generic point (yellow circle) on the luminal surface at different time points (grey arrows) along the cardiac cycle, concurring to *TAWSS* determination in that point. Blue and red arrows indicate the WSS vector field instantaneous streamlines in the surroundings of the generic point (yellow circle) on the luminal surface at different time points along the cardiac cycle, concurring to *TSVI* determination in that point.

Explanatory examples of different flow environments at the luminal surface of an artery are depicted in Fig. 3 aiming to highlight differences between *TSVI* and *TAWSS* biomechanical significance. In particular, the figure shows that: (1) the *TAWSS* value computed on a point at the luminal surface depends solely on the magnitude of the WSS vector applied to such a point along the cardiac cycle; (2) the *TSVI* value computed at a point depends on the instantaneous WSS vector field configuration in a neighbourhood of such a point along the cardiac cycle. The WSS-based quantities were averaged over the same 3 mm/45 degrees sectors at the luminal surface as the WT data.

Finally, as suggested by evidences on the atheroprotective role played by helical blood flow,^10^,^16^ a possible link between the WSS topological skeleton and helical flow features was qualitatively investigated. The intention here was to clarify whether WSS topological skeleton features reflect the presence of intravascular flow patterns inversely associated with WSS^11^ and WT in coronary arteries.^10^ To do that, the local normalized helicity (*LNH*), representing the cosine of the angle between local velocity and vorticity vectors, was used to visualize right- and left-handed helical blood flow patterns (positive and negative *LNH* values, respectively^16^) inside the coronary artery models.^11^

### Statistical Analysis

Differences among the occurrence of the instantaneous WSS fixed points (i.e., saddle points, sources, and sinks) at the coronary luminal surface over the cardiac cycle were evaluated with a Wilcoxon signed-rank test. The existence of possible relations between near-wall hemodynamic features and measured WT data was investigated in terms of odds ratio, a measure of the strength of the association between two events. More in detail, we classified as hemodynamically relevant the luminal surface sectors exposed to low or high values of the WSS-based hemodynamic descriptors *via* quintiles, identifying, as artery-specific thresholds, the 20th and the 80th percentiles of the WSS-based descriptors distribution, respectively. The combination of WSS-based descriptors pairs was also investigated by considering the luminal surface sectors where both descriptors were classified as hemodynamically relevant. To determine the odds ratio, coronary artery WT and WT growth rate values were divided into low, mid or high based on a vessel-specific tertiles division.^10^,^11^,^21^ Statistical significance was assumed for *p *< 0.05.

## Results

The number and nature of instantaneous WSS fixed points at the coronary luminal surface were analysed and compared at time points T1 and T2 (Figure S2 of the Supplementary Materials). As a main result, we report here that comparing the prevalence of the instantaneous WSS fixed points of the same nature at T1 and T2, no significant differences emerged (saddle points *p *= 0.47, sinks *p *= 0.44, sources *p *= 0.39). Therefore, it is expected that the overall structure of the WSS topological skeleton does not markedly change its spatial configuration in the time interval T2-T1, as the number and nature of fixed points affect the pattern of manifolds onto the luminal surface.

**Figure 4:**
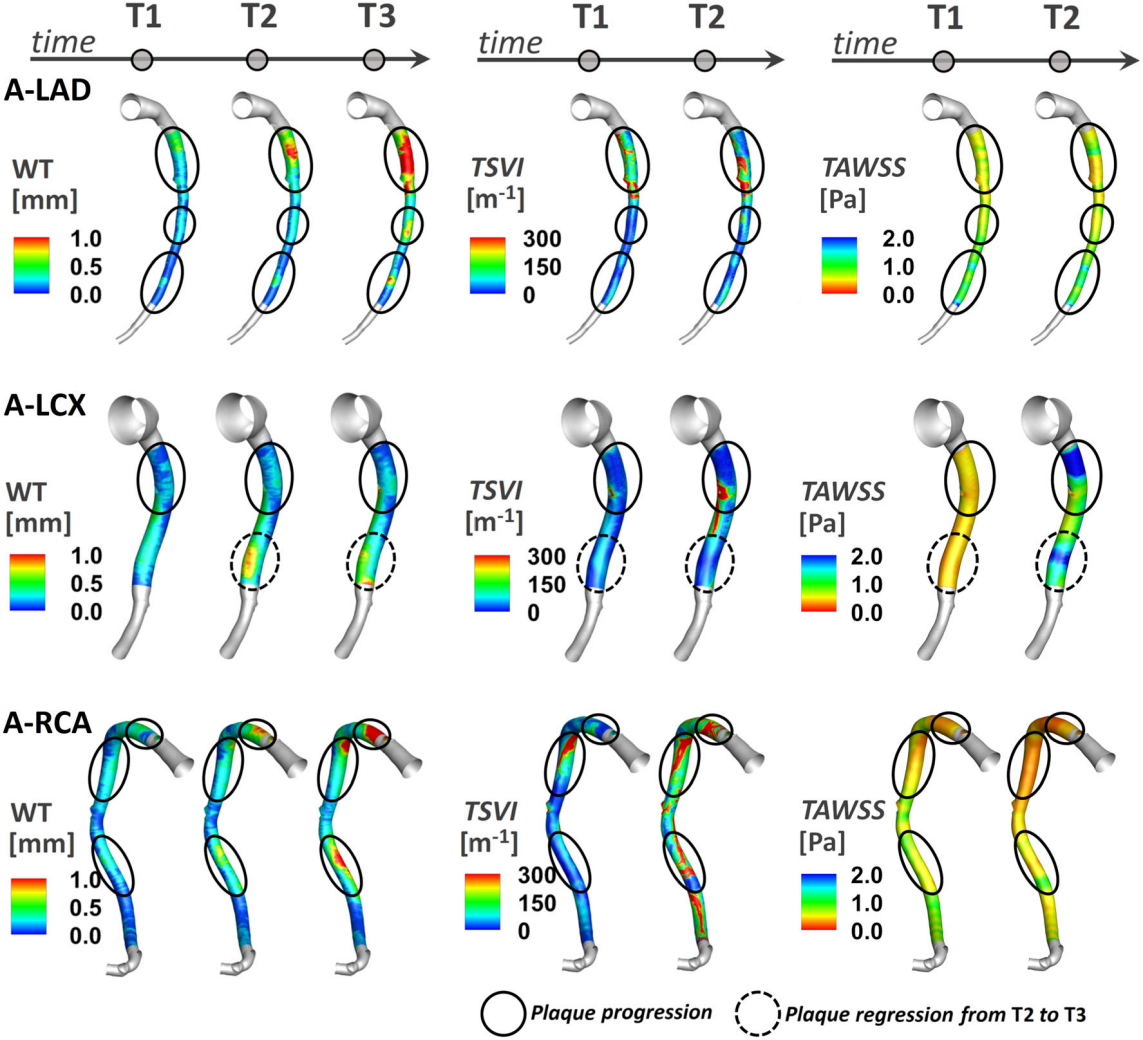
Luminal distributions of measured WT, *TSVI*, and *TAWSS* at several time points along the follow-up time for the coronary artery models of pig A. The distributions of WT, *TSVI*, and *TAWSS* are only shown along the IVUS segment of the main vessel. The side branches are not shown. The regions of interest identified along each vessel are emphasized by solid black circle if experiencing plaque progression over time or dashed black circle if experiencing plaque regression between T2 and T3.

The variation of the WSS contraction/expansion action along the cardiac cycle, expressed in terms of *TSVI* luminal distribution at T1 and T2, is presented in Fig. 4 for three coronary models (LAD, LCX, and RCA of pig A in Figure S1 of the Supplementary Materials) together with the distribution of measured WT at time points T1, T2 and T3. It can be observed that in general: (1) luminal surface regions exposed to high *TSVI* values co-located with high WT values; (2) luminal surface areas exposed to high *TSVI* values at T1 co-locate with regions of WT progression in the time interval T2-T1 and T3-T1; (3) albeit moderately, luminal surface areas experiencing an increase in *TSVI* from T1 to T2 co-locate with regions of WT growth over time (as can be seen along the LAD and RCA, and at the proximal part of LCX shown in Fig. 4); (4) albeit moderately, luminal surface areas experiencing a decrease in *TSVI* from T1 to T2 co-locate with regions of WT regression in the time interval T3–T2 (see e.g., the distal part of LCX in Fig. 4). In general, the reported observations are common to all the coronary models involved in the study (Figure S3 of the Supplementary Materials).

From a parallel analysis of *TAWSS* luminal distribution at T1 and T2 (Fig. 4), it can be observed that in general: (1) luminal surface regions exposed to low *TAWSS* values present with high WT values; (2) luminal surface areas experiencing low *TAWSS* values at T1 present with WT values at T2 and T3 higher than at T1; (3) luminal surface areas exposed to an increase in *TAWSS* from T1 to T2 only partially co-localize with regions of WT regression in the time interval T3-T2 (see e.g., the distal part of LCX of pig A in Fig. 4).

### Link Between Wall Shear Stress and High Wall Thickness (Adverse Outcome)

**Figure 5:**
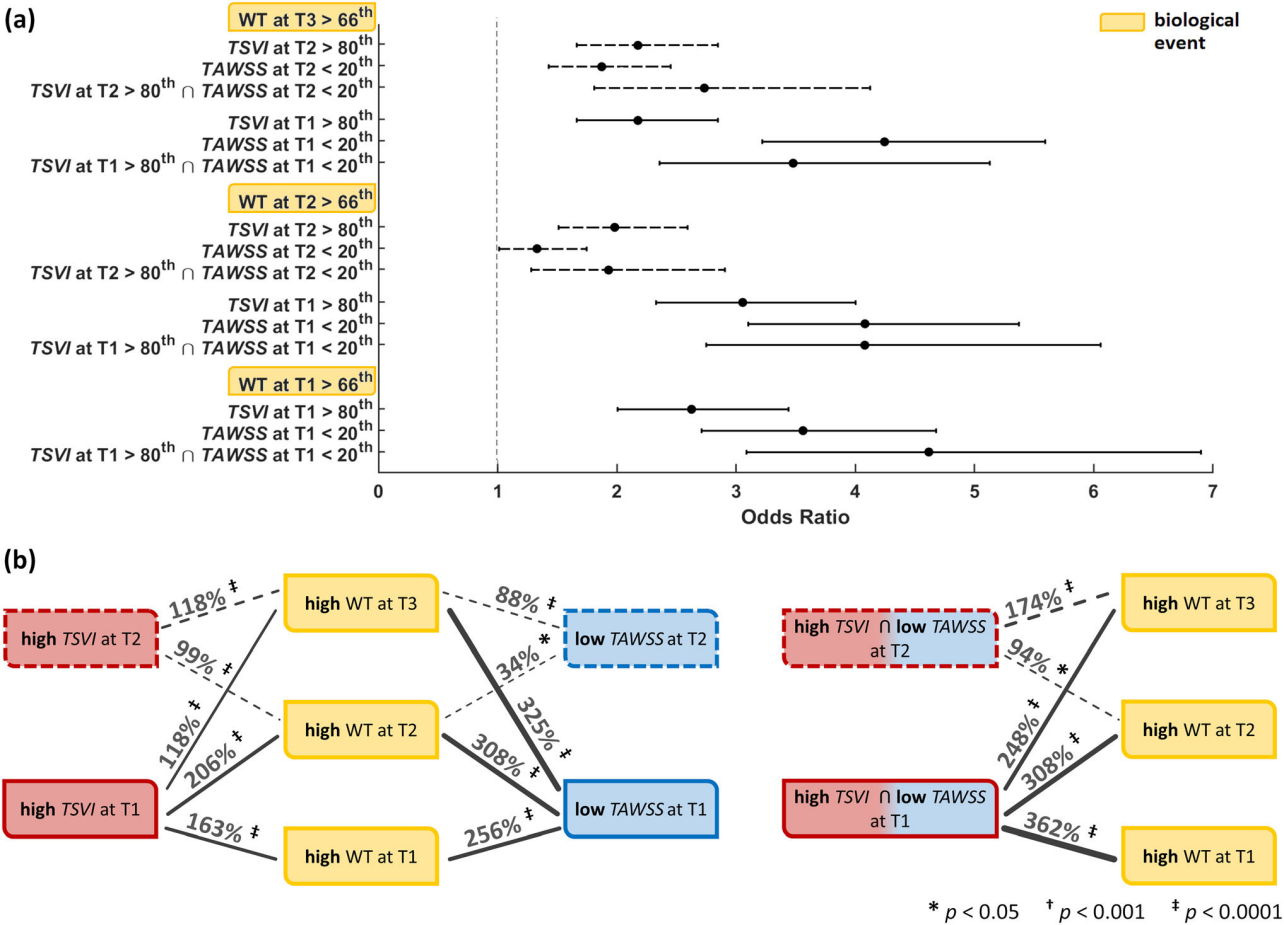
Predictive ability of hemodynamic descriptors for high WT. (a): odds ratios with associated confidence interval for hemodynamic events vs. high WT at the various time points (b): graphical sketch of the identified significant associations between hemodynamic and biological events. The adverse (high WT) biological events are indicated in yellow. Red and blue indicate (deleterious) high *TSVI* and low *TAWSS*, respectively. Associations are represented by black lines whose thickness is proportional to the indicated percent increase in odds of high WT (*p*-values also shown). Solid vs. dashed lines indicate associations for hemodynamic events at T1 vs. T2, respectively.

The analysis is presented in terms of odds ratio (Fig. 5a, and Table S2 of the Supplementary Materials) as well as in terms of percentage increase in the odds of the adverse outcome (Fig. 5b). For this analysis, high WT (> 66th percentile) was considered as the adverse outcome. The exposure to high *TSVI* (> 80th percentile) or to low *TAWSS* (< 20th percentile) largely predicted the same connections to adverse outcome (Fig. 5, and Table S2 of the Supplementary Materials). Looking at the data longitudinally first, both low *TAWSS* and high *TSVI* at *earlier* time points were good predictors of *later* thickening. Specifically, low *TAWSS* was a slightly stronger predictor than high *TSVI* (increase in the odds of later adverse outcome in the ranges of 88-325 and 99-206%, respectively), except at T2, where high *TSVI* was stronger predictor of high WT at T3. The combination of low *TAWSS* and high *TSVI* was an even stronger predictor of adverse outcome at T3 (174% increase in the odds) than low *TAWSS* or high *TSVI* alone (88 and 118% increase in the odds, respectively, Fig. 5). Now looking at the data transversally, both low *TAWSS* and high *TSVI* at T1 and T2 also co-localized with high WT at their respective time points, with low *TAWSS* more strongly co-localized at T1 (256 vs. 163% of increase in the odds) and high *TSVI* at T2 (99 vs. 34% of increase in the odds). Locations experiencing contemporaneous low *TAWSS* and high *TSVI* at T1 presented with increased odds of high WT at T1 (362%), markedly higher than single quantities (256 and 163% increase in the odds, respectively, Fig. 5).

### Link Between Wall Shear Stress and Low Wall Thickness (Favourable Outcome)

**Figure 6:**
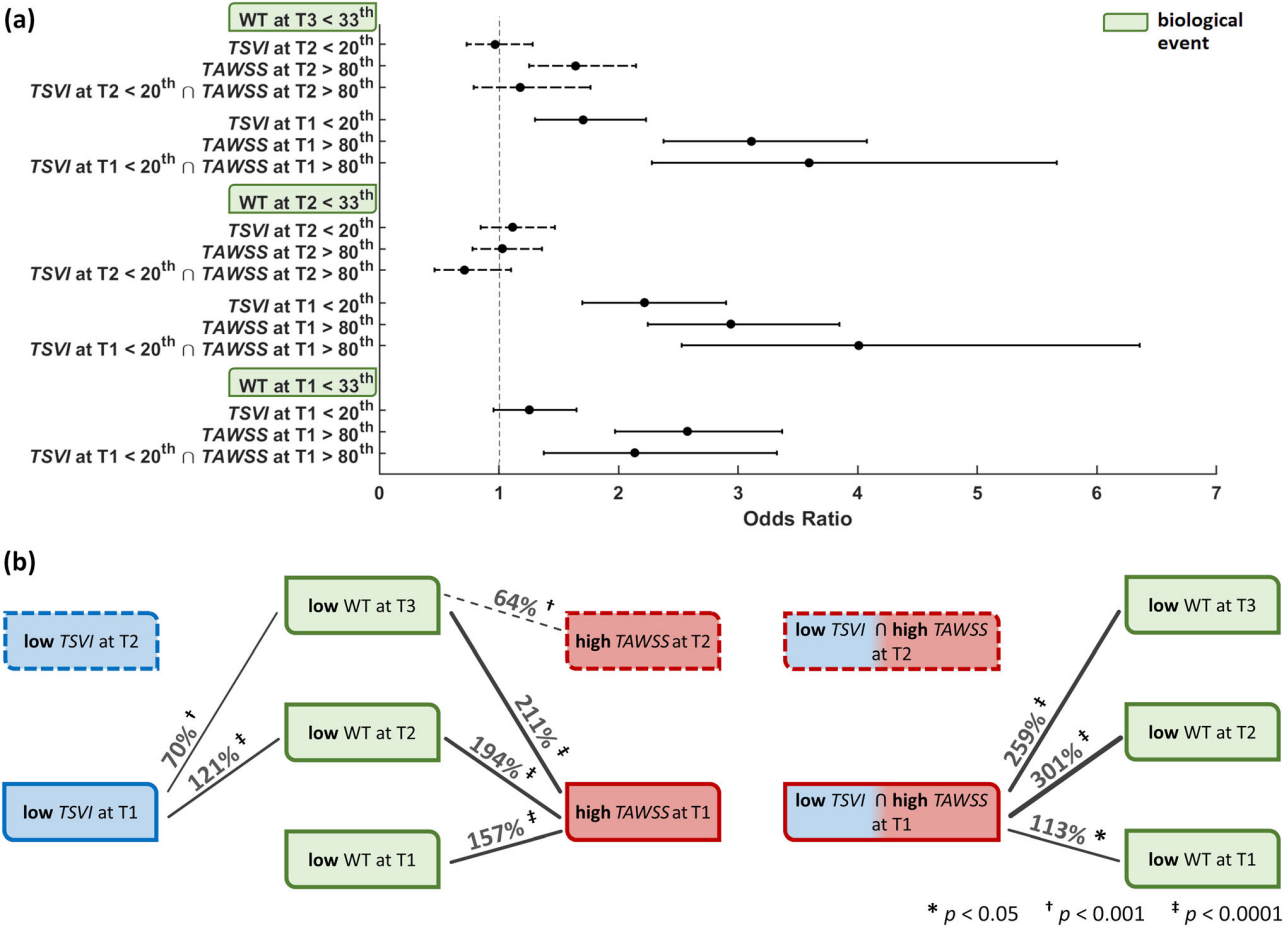
Predictive ability of hemodynamic descriptors for low WT. (a): odds ratios with associated confidence interval for hemodynamic events vs. low WT at the various time points (b): graphical sketch of the identified significant associations between hemodynamic and biological events. The favourable (low WT) biological events are indicated in green. Blue and red now indicate (ostensibly physiologically-normal) low *TSVI* and high *TAWSS*, respectively. Associations are represented by black lines whose thickness is proportional to the indicated percent increase in odds of high WT (*p*-values also shown). Solid vs. dashed lines indicate associations for hemodynamic events at T1 vs. T2, respectively.

Mirroring the analysis for adverse outcome, here we applied the same approach to favourable outcome, defined as low WT (< 33rd percentile). The exposure to low *TSVI* (< 20th percentile) or to high *TAWSS* (> 80th percentile) and their association with low WT is presented in Fig. 6 (see also Table S3 of the Supplementary Materials). From the longitudinal analysis it emerged that high *TAWSS* at *earlier* time points was a stronger predictor of favourable outcome than low *TSVI* (increase in the odds of later favourable outcome in the ranges of 64–211 and 70–121%, respectively). To be noted, low *TSVI* at T2 was not a strong predictor of low thickening at T3 (Fig. 6). The combination of high *TAWSS* and low *TSVI* at T1 was an even stronger predictor of favourable outcome (301 and 259% increase in the odds at T2 and T3, respectively) than low *TAWSS* or high *TSVI* alone (Fig. 6). The same combination at T2 was unable to predict low thickening at T3. Looking at the data transversally, only low *TAWSS* at T1 co-localized with low WT at the same time point (157% increase in the odds of favourable event, Fig. 6). Locations experiencing contemporaneous high *TAWSS* and low *TSVI* at T1 presented with increased odds of low WT at the same time point (113%), but lower than single quantity high *TAWSS* (113 vs. 157% increase in the odds, respectively, Fig. 6).

### Link Between Wall Shear Stress Topological Skeleton and Intravascular Hemodynamics

**Figure 7:**
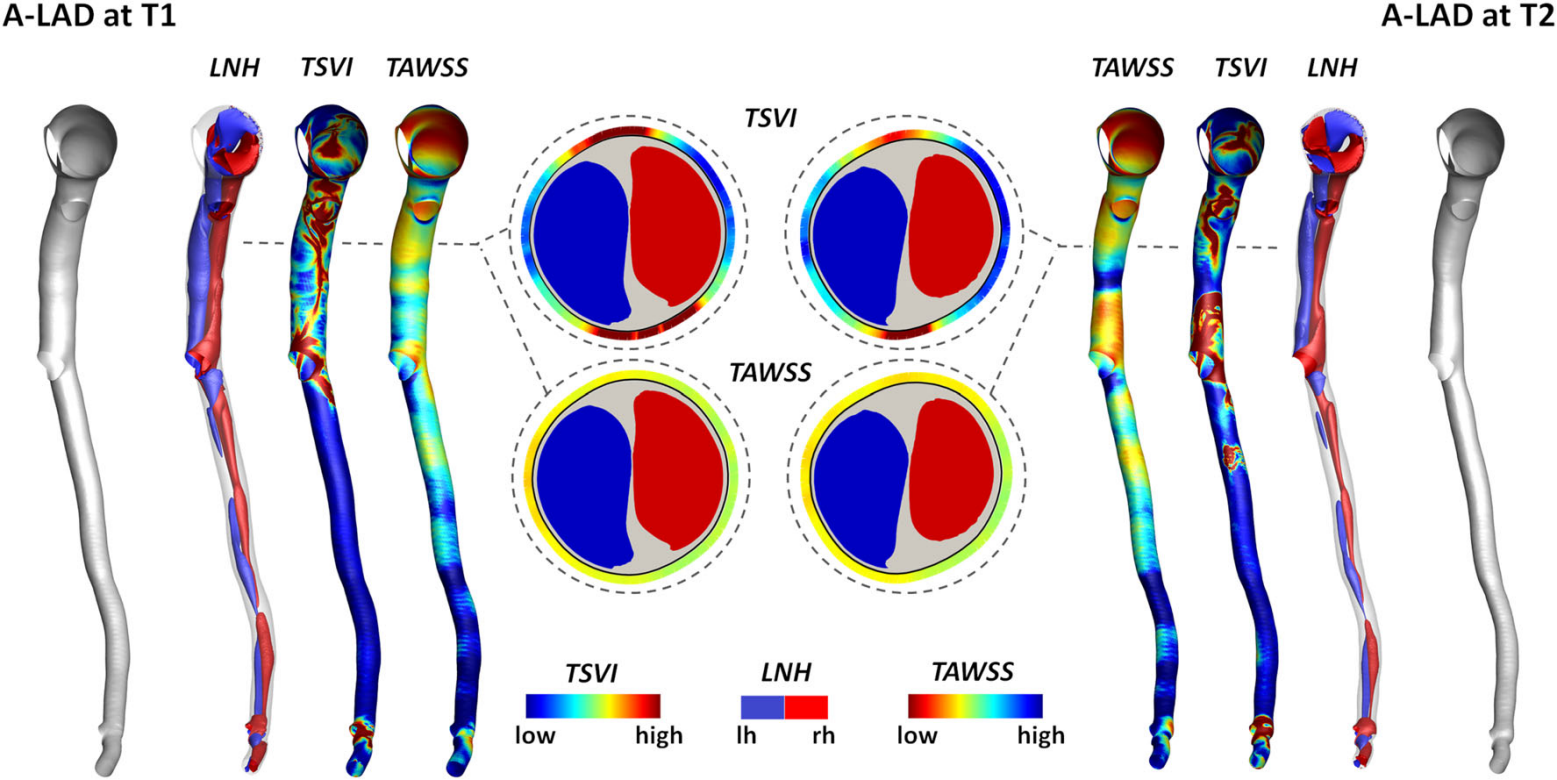
Distribution of intravascular helical blood flow features and *TSVI* and *TAWSS* at the luminal surface of the LAD of pig A at T1 (left) and T2 (right) of the study. Right-handed (*LNH *> 0) and left-handed (*LNH *< 0) helical blood structures are displayed by red and blue, respectively. The luminal distributions of *TSVI* and *TAWSS* are also shown. Only the main vessel is displayed; the side branches are not depicted. One explanatory luminal cross-section per model is reported. The lumen contour is markedly thick for a better visualization of *TSVI* and *TAWSS* values at the luminal cross-section edge.

To clarify whether WSS topological skeleton features reflect intravascular helical flow patterns, which have been shown to have an atheroprotective role in coronary arteries,^10^,^11^ an explanatory coronary model (LAD of pig A) is presented in Fig. 7. By visual inspection, it emerged that at time T1 and T2 the luminal surface areas experiencing high variation in the WSS contraction/expansion action along the cardiac cycle (expressed in terms of *TSVI*) enclosed the separation region between counter-rotating helical blood flow patterns (highlighted by red and blue coloured isosurfaces of cycle-average *LNH* values in Fig. 7). The latter is emphasized by displaying *TSVI* and *LNH* (and, for sake of completeness, *TAWSS*) distributions at explanatory luminal cross-sections along both the proximal and distal arterial segments in Fig. 7. In general, those observations were common to all the coronary models involved in the study.

## Discussion

In previous studies, coronary atherosclerotic plaque development has been linked to local time-varying hemodynamic features, such as the local WSS along the cardiac cycle, oscillatory changes in its direction and multidirectionality.^12^,^28^,^36^,^47^ Currently, it is unclear which are the consequences of the spatial configuration of the shear forces acting in the neighbourhood of a given point, i.e., as opposed to pointwise WSS features, in terms of atherosclerosis initiation/progression. The spatial configuration patterns can be interpreted as a contraction/expansion action exerted by the shear forces acting on the endothelium, or, in other words, a push/pull action exerted by the WSS on the endothelium. These patterns can be identified by the WSS vector field divergence, which represents the rate of separation of close WSS trajectories. In terms of biomechanics, the contraction/expansion action exerted by shear forces on ECs is responsible for both intracellular and intercellular tension, which in turn may impact on, e.g., shrinking/widening of cellular gaps, ultimately leading to wall thickening due to atherosclerosis.^24^,^29^

### Summary of Findings

The most striking results to emerge from this study are that (per Figs. 5, 6, and 7, and Tables S2 and S3 of the Supplementary Materials): (1) both investigated hemodynamic stimuli at the endothelial level—high variations in the WSS contraction/expansion action on the endothelium along the cardiac cycle (quantified by *TSVI*) and low time-average WSS magnitude (quantified by *TAWSS*)—were associated with WT growth, accounting for different hemodynamic effects portending atherosclerosis; (2) *TSVI* and *TAWSS* values in a physiological range appeared to play an atheroprotective role on ECs; (3) high variations in WSS manifolds dynamics along the cardiac cycle corresponded to intravascular regions of separation between left- and right-handed helical flow patterns.

The present findings expand the impact of local hemodynamics on the onset/development of coronary atherosclerosis, suggesting that, in addition to low WSS,^27^,^44^,^47^ also the high variability in the action exerted by the WSS in the neighbourhood of a generic position at the luminal surface may represent a relevant hemodynamic cue in coronary artery disease. Results from this study indicate that the WSS manifolds dynamics along the cardiac cycle—here quantified in terms of *TSVI*—is linked to WT in coronary arteries, with a relationship with future WT patterns. This relation is explained by the fact that WSS manifolds dynamics along the cardiac cycle reflects those near-wall flow disturbances^2^,^33^ that are known to condition the natural history of coronary atherosclerosis.^36^

### Biological Mechanisms Linking *TSVI* to Wall Thickness

This study confirms recently emerged evidence that WSS topological skeleton features influence vascular pathophysiology.^12^,^37^ The main novelty of the study, i.e., the emerged association of *TSVI* with WT, can be interpreted by linking biomechanics to possible biological mechanisms involved in atherosclerosis. In detail, on the one hand an ostensibly *normal* variability of the contraction/expansion action exerted by the WSS on the endothelium during the cardiac cycle is expected to maintain ECs in a quiescent state, with a fusiform shape and with stable intercellular junctions,^5^,^7^,^47^ stimulating the expression of atheroprotective and antithrombogenic genes contributing to prevent atherosclerotic plaque initiation.^7^ On the other hand, the luminal exposure to *markedly variable* WSS manifolds dynamics during the cardiac cycle may mechanically induce recurring variations in ECs intracellular as well as intercellular tensions (e.g. acting on cell junctions inclinations),^9^,^22^,^29^,^35^,^38^ with consequent alteration of the mechanism of tension propagation not only within the cell, but also from cell to cell. The latter may modulate ECs translation and transcription, thus affecting their pro-atherogenic susceptibility.^22^ A plausible consequence for changes in directionality and magnitude of cellular tensions is, indeed, the shrinking/widening of ECs junctions, which influences the endothelium permeability to atherogenic species such as lipoproteins,^3^,^7^,^46^ and/or promotes endothelium proinflammatory phenotype enhancing EC oxidative state.^7^,^19^ Moreover, it may impact ECs quiescence and obstruct physiological ECs fusiform shaping. On top of that, in turn, all these biological mechanisms are recognized to stimulate EC atherogenic genes expression as well, thus potentially promoting atherosclerosis initiation and faster progression.^3^,^5^,^7^,^46^,^47^ Although the luminal exposure to high *TSVI* may induce similar pro-atherogenic biological effects compared to low *TAWSS*, the two hemodynamic events are different from a mechanistic viewpoint (as sketched in Fig. 3).

### Making Sense of Hemodynamic Features with Respect to Coronary Atherosclerosis

The widely recognized role played by WSS magnitude (biomechanically described by the canonical indicator *TAWSS*) in coronary atherogenesis and development^31^,^44^,^47^ was here confirmed: high WT was observed along the follow-up time at luminal sectors exposed to low *TAWSS* (Fig. 5, and Table S2 of the Supplementary Materials). However, according to previous studies revealing its weak-to-moderate ability to predict lesion localization and development^27^,^40^,^44^ or endothelial dysfunction^15^ at the early stage, our findings suggest that low *TAWSS* *per se* does not fully characterize the WSS features promoting coronary atherosclerosis onset/progression. A possible explanation for this evidence may have to do with the mechanistic meaning of *TAWSS* that, *stricto sensu*, measures the local cycle-average WSS magnitude and thus its low value reflects (1) a locally weak shear force on the endothelium and, in general, (2) a thickened diffusive boundary layer for blood-wall mass transfer.

**Figure 8:**
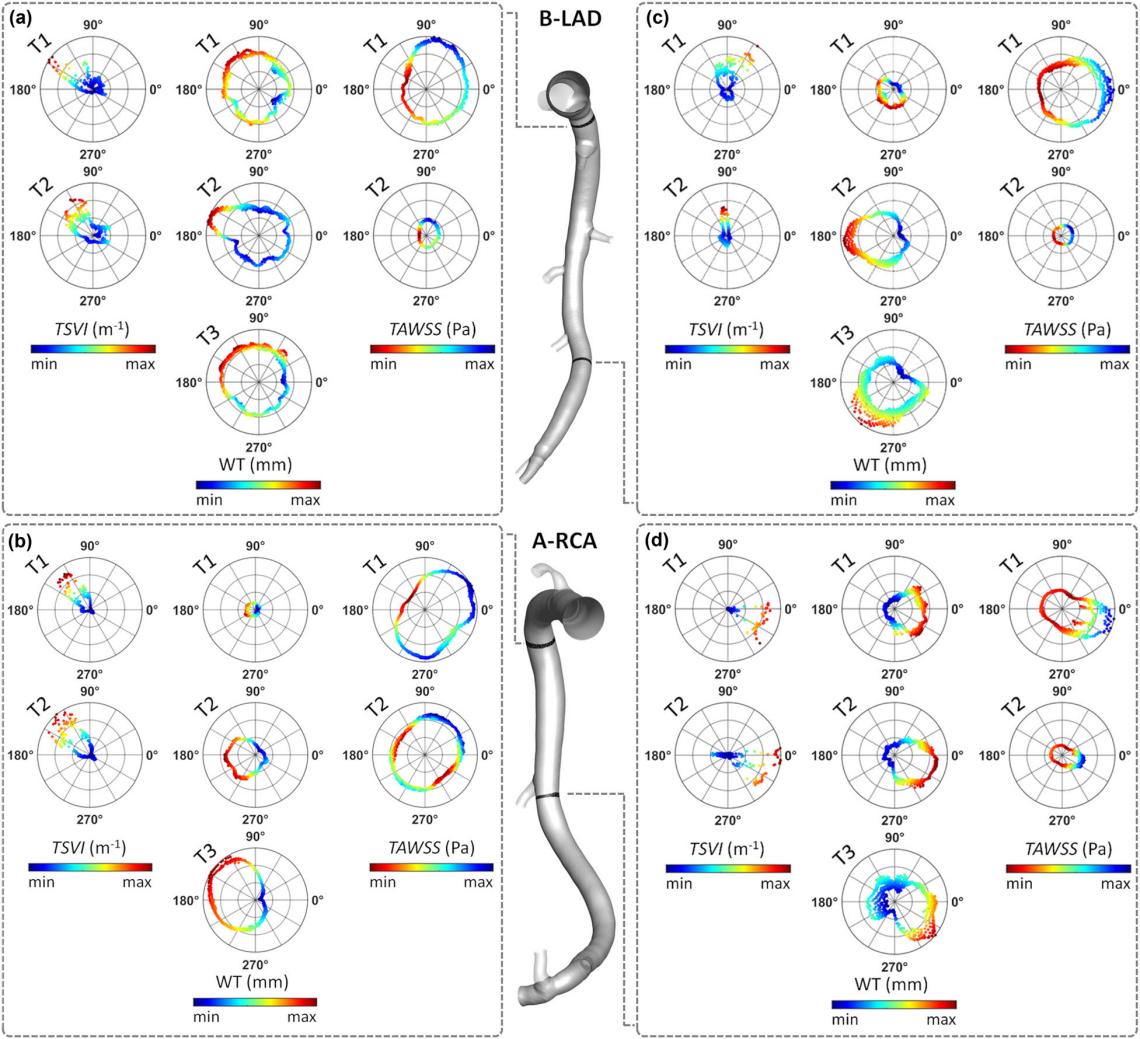
Polar plots showing *TSVI*, WT, and *TAWSS* cross-sectional distributions at several time points along the follow-up time for the LAD of pig B (top) and the RCA of pig A (bottom). The distributions of *TSVI*, WT and *TAWSS* over the follow-up time are shown at two explanatory cross-sections per vessel (coloured by black) along the IVUS-imaged segment. Colormaps are normalized by the maximum value of each descriptor.

As explained with examples reported in Fig. 3 and as expected by its definition, *TSVI*, reflecting the variability of the fluid forces generating endothelial intracellular and intercellular tensions, allows the identification of peculiar flow disturbances linked to biological events that cannot be captured by low *TAWSS*. A confirmation is given by the observation that, considering the luminal sectors where high *TSVI* or low *TAWSS* co-localized with high WT, the co-localization of the two WSS-based metrics was observed in only the 14.2% of the sectors. Therefore, low *TAWSS* and high *TSVI* account for different hemodynamic effects, both linked to WT changes. In support of this, the explanatory examples presented in Fig. 8 for two different vessels, where polar plots were used to analyse the cross-sectional distribution of WT, *TAWSS* and *TSVI* as well as the observable co-localization for those quantities. In detail, Fig. 8 shows the cases when: (1) high *TSVI* and low *TAWSS* co-localize with high WT (Fig. 8, panels a, b); (2) low *TAWSS* only co-localizes with high WT (Fig. 8, panel c); (3) high *TSVI* only co-localizes with high WT (Fig. 8, panel d). Therefore, the analysis in Fig. 8 further supports the hypothesis that high *TSVI* is to be intended as not in competition with low *TAWSS*, but as able to integrate the characterization of the WSS features involved in the onset/progression of coronary atherosclerosis.

Of note, the findings of this study also confirm the atheroprotective role of physiological WSS, while suggesting that also physiological values of *TSVI* protect the coronary arteries from plaque progression (Fig. 6).

Finally, the findings of this study highlight that a link exists between the luminal *TSVI* distribution and intravascular helical flow patterns (Fig. 7), since high *TSVI* values were in general found in correspondence of the separation between counter-rotating helical structures. This recapitulates the known beneficial influence of helicity in suppressing flow disturbances in main arteries^16^ and, in particular, its atheroprotective role in the context of coronary atherosclerosis.^10^,^11^ Furthermore, these findings enforce previous suggestions on the use of helical flow-based quantities as *in vivo* surrogate marker of atherosclerotic plaque onset/progression in coronary arteries.^10^ In the next future, the advances in phase-contrast magnetic resonance imaging are expected to make the *in vivo* quantification of arterial helical flow feasible also in small vessels,^32^ as coronary arteries, allowing the non-invasive *in vivo* prediction of atherosclerosis progression based on helical flow descriptors.

### Potential Limitations of the Study

In the numerical simulations, vascular walls were assumed to be rigid. However, it has been reported that the vascular wall elasticity does not markedly affect the distribution of canonical WSS-based quantities,^14^ and we expect a similar result for *TSVI*. In addition, the cardiac-induced motion of coronary arteries was neglected, based on previous findings demonstrating the moderate effect of myocardial motion on coronary flow and WSS distribution.^49^ Measured flow rates were imposed in terms of time-dependent flat velocity profile. It has been recently demonstrated that imposing measured ComboWire Doppler-based flow rates in terms of flat velocity profile at the inflow boundary of computational models of coronary arteries moderately influence the luminal distribution of the near-wall hemodynamics only at the most proximal segment of each coronary artery model.^30^ For this reason, such an assumption is here expected to have a very minor impact, as the analysis is focused on the IVUS-imaged segment of each model, the most proximal coronary segment being not considered. The generality of the presented study might be limited by the small number of coronary artery models used in the analysis (*N* = 9). However, here we considered multiple sectors within each coronary artery, and this allowed for statistically significant relations to emerge, revealing links between local hemodynamics and WT. Finally, our findings are based on a pig model; however, such models, because of the similar anatomy with human coronary arteries, are widely used in studies of coronary artery diseases.^48^

In conclusion, the present study confirms the existence of links between WSS topological skeleton and markers of early vascular disease in coronary arteries, contributing to a more accurate depiction of the role of WSS in vascular pathophysiology. In general terms, the findings from this work suggest that the quantified variability in the contraction/expansion action exerted by the WSS on the endothelium along the cardiac cycle (1) is associated with the progression/regression of biologically relevant vascular events, and (2) represents a hemodynamic cue that well-established local WSS-based descriptors are unable to fully capture. Therefore, in addition to low WSS, the variability in the action exerted by WSS at the arterial luminal surface contributes to a deeper understanding of the hemodynamic-related impact on the onset/development of coronary atherosclerosis.

### Supplementary Information

Below is the link to the electronic supplementary material.

Supplementary material 1 (PDF 1015 kb)

## Figures and Tables

**Figure 9:**
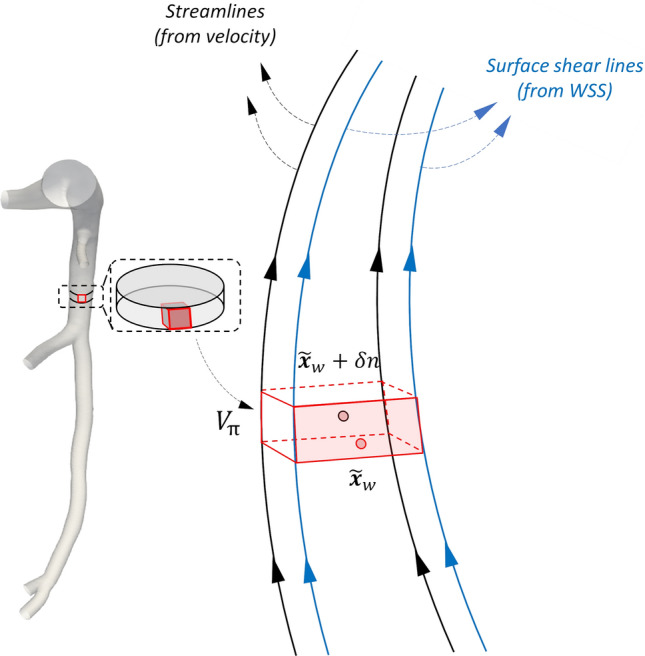
Explanatory sketch of an elemental volume $V_{\pi } \left( t \right)$ near the wall. ${\user2{\tilde{x}}}_{w}$ is a point on the luminal surface and ${\user2{\tilde{x}}}_{w} + \delta n$ is a point inside the volume $V_{\pi } \left( t \right)$ such that the distance between the two points in the direction normal to the wall is $\delta n.$ Blue and black lines represent surface shear lines and streamlines of velocity, respectively.

## Appendix

The use of the normalized WSS divergence to identify WSS manifolds has been recently proposed for Newtonian fluids.^33^ Here, we extend the applicability of the WSS divergence-based method for the identification of WSS manifolds to the class of Reiner-Rivlin fluids, also called generalised Newtonian fluids.^42^

Let *Ω* ⊂ ${\mathbb{R}}^{3}$ be a bounded domain representing the lumen of a vessel. For each position ${\user2 {x}} \in \varOmega$ and time $t \in {\mathbb{R}}^{ + }$, we denote by ${ \user2{u}}_{\user2{\pi }} \left( {\user2{x},t} \right)$ the near-wall tangential velocity, i.e., the tangential component of the velocity close to the luminal surface. It has been demonstrated elsewhere^17^ that the component of the velocity normal to the wall is second order in $\delta n$, where $\delta n$ represents the intravascular normal distance from the wall. Therefore, it can be considered to have lesser degree of importance in the analysis. Let $V_{\pi } \left( t \right)$ be an elemental volume near the wall and $S\left( t \right) = \delta V_{\pi } \left( t \right)$ be a closed surface enclosing the volume $V_{\pi } \left( t \right)$, as depicted in Fig. 9.

The rate of near-wall volume variation can be expressed as:

A1 $$\frac{{dV_{\pi } \left( t \right)}}{{dt}} = \iint_{{S\left( t \right)}} {{ \user2{u}}_{\user2{\pi }} {\text{ }} \cdot \user2{n} \, dS} = \iiint_{{V_{\pi } \left( t \right)}} {\nabla {\text{ }} \cdot { \user2{u}}_{\user2{\pi }} \, dV_{\pi } },$$

where $\varvec{n}$ is the unit normal to the surface $S.$ Now let ${\user2{\tilde{x}}}_{w} \in \delta \varOmega$ be a point on the luminal surface and let ${\user2{\tilde{x}}}_{w} + \delta n \in \varOmega$ be a point inside the volume $V_{\pi } \left( t \right)$, such that the distance between the two points in the direction normal to the wall is $\delta n .$ Shrinking the near-wall volume $V_{\pi }$ to zero, we obtain:

A2 $$\mathop {\lim }\nolimits_{{V_{\pi } \to 0}} \frac{1}{{V_{\pi } }}\frac{{dV_{\pi } \left( t \right)}}{{dt}} = \mathop {\lim }\nolimits_{{V_{\pi } \to 0}} \frac{1}{{V_{\pi } }}\iiint_{{V_{{\pi \left( t \right)}} }} {\nabla \cdot { \user2{u}}_{\user2{\pi }}\, dV_{\pi } } = \left( {\nabla \cdot { \user2{u}}_{\user2{\pi }} } \right)|_{{{\user2{\tilde{x}}}_{w} + \delta n}} .$$

Using a Taylor series expansion in space of the near-wall tangential velocity, it follows that:

A3 $${ \user2{u}}_{\user2{\pi }} \left( {{\user2{\tilde{x}}}_{w} + \delta n,t} \right) \approx { \user2{u}}_{\user2{\pi }} \left( {{\user2{\tilde{x}}}_{w} ,t} \right) + \left( {{\user2{\tilde{x}}}_{w} + \delta n - {\user2{\tilde{x}}}_{w} } \right)\frac{{\delta { \user2{u}}_{\user2{\pi }} }}{\delta n}|_{{{\user2{\tilde{x}}}_{w} }} + O\left( {\delta n^{2} } \right) \approx { \user2{u}}_{\user2{\pi }} \left( {{\user2{\tilde{x}}}_{w} ,t} \right) + \left( {\delta n} \right)\frac{{\delta { \user2{u}}_{\user2{\pi }} }}{\delta n}|_{{{\user2{\tilde{x}}}_{w} }} + O\left( {\delta n^{2} } \right),$$

where ${ \user2{u}}_{\user2{\pi }} \left( {{\user2{\tilde{x}}}_{w} ,t} \right)$ is equal to zero due to the non-slip condition.

When assuming a generalized Newtonian behavior for the fluid, the dynamic viscosity $\mu$ is dependent on the shear rate $\dot{\gamma }$, defined as:

A4 $$\dot{\gamma } = \sqrt {2\user2{D}\left( \user2{u} \right):\user2{D}\left( \user2{u} \right) } = \sqrt {2tr \left( \user2{D} ( {\user2{u})^{2} } \right) } ,$$

where $\user2{D}\left( \user2{u} \right) = \frac{{\nabla \user2{u} + \nabla \user2{u}^{T} }}{2}$ is the rate of deformation tensor and (:) indicates the double dot product. Based on the definition of the WSS for a generalized Newtonian fluid^42^:

A5 $${\user2{\tau }} = \mu \left( {\dot{\gamma }} \right)\left( {\frac{{\delta { \user2{u}}_{\user2{\pi }} }}{\delta n}} \right)|_{n = 0} ,$$

it can be obtained that:

A6 $$\frac{{\delta { \user2{u}}_{\user2{\pi }} }}{\delta n}|_{{{\user2{\tilde{x}}}_{w} }} = \frac{{\user2{\tau }} }{{\mu \left( {\dot{\gamma }} \right)}}|_{{{\user2{\tilde{x}}}_{w} }} .$$

Substituting Eq. (7) into Eq. (4), it follows that:

A7 $${ \user2{u}}_{\user2{\pi }} \left( {{\user2{\tilde{x}}}_{w} + \delta n,t} \right) \approx \frac{\delta n}{{\mu \left( {\dot{\gamma }} \right)}} {\user2{\tau }} \left( {{\user2{\tilde{x}}}_{w} ,t} \right) + O\left( {\delta n^{2} } \right).$$

Considering the approximation of the near-wall tangential velocity (Eq. (A7)) in Eq. (A2), it follows that:

A8 $$\left( {\nabla \cdot { \user2{u}}_{\user2{\pi }} } \right)|_{{{\user2{\tilde{x}}}_{w} + \delta n}} \approx \left( {\nabla \cdot\frac{\delta n}{{\mu \left( {\dot{\gamma }} \right)}}{\user2{\tau }} } \right)|_{{{\user2{\tilde{x}}}_{w} }} \approx \delta n\left( {\nabla \cdot \frac{{\user2{\tau }} }{{\mu \left( {\dot{\gamma }} \right)}}} \right)|_{{{\user2{\tilde{x}}}_{w} }} .$$

It is evident that Eq. (1) can be also expressed as follows:

A9 $$\nabla \cdot \left( {\frac{{\frac{{\user2{\tau }} }{{\mu \left( {\dot{\gamma }} \right)}}}}{{\parallel{\frac{{\user2{\tau }} }{{\mu \left( {\dot{\gamma }} \right)}}}\parallel_2 }}} \right) = \nabla \cdot \left( { \frac{{\user2{\tau }} }{\parallel {\user2{\tau }} \parallel_2}} \right) = DIV_{\text{WSS}} ,$$

meaning that the use of the quantity used in the Eulerian-based approach to identify WSS contraction/expansion regions (Eq. (1))^33^ can be extended to the analysis of the WSS topological skeleton of a generalised Newtonian fluid.
