# Supplementary material for: Early Atherosclerotic Changes in Coronary Arteries are Associated with Endothelium Shear Stress Contraction/Expansion Variability
Source: Ann Biomed Eng. 2021 Jul 29;49(9):2606–21. doi: 10.1007/s10439-021-02829-5 (PMC8455396; doi:10.1007/s10439-021-02829-5)
Supplement: Supplementary file 1 — Supplementary material 1 (PDF 1015 kb) [file 10439_2021_2829_MOESM1_ESM.pdf]

SUPPLEMENTAL MATERIAL

Early atherosclerotic changes in coronary arteries are associated with endothelium shear stress contraction/expansion variability

Valentina Mazzi[1*], Giuseppe De Nisco[1*], Ayla Hoogendoorn[2], Karol Calò[1], Claudio Chiastra[1], Diego Gallo[1], David A. Steinman[3], Jolanda J. Wentzel[2], Umberto Morbiducci[1†]

[1] *PoliTo[BIO]Med Lab, Department of Mechanical and Aerospace Engineering, Politecnico di Torino, 10129 Turin, Italy*

[2] *Department of Cardiology, Biomedical Engineering, Erasmus MC, 3000 CA Rotterdam, The Netherlands*

[3] *Biomedical Simulation Laboratory, Department of Mechanical & Industrial Engineering, University of Toronto, Toronto, Canada*

[*]The authors equally contributed to this study

[†]**Address for correspondence**:

Umberto Morbiducci, Ph.D.

Department of Mechanical and Aerospace Engineering, Politecnico di Torino

Corso Duca degli Abruzzi, 24 - 10129 Turin, Italy

Tel.: +39 011 0906882

Fax: +39 011 5646999

E-mail: umberto.morbiducci@polito.it

Supplemental Methods

Medical Imaging and Geometry Reconstruction

An in-house developed algorithm was used to trigger offline intravascular ultrasound (IVUS) images and to remove the heartbeat artefact. The coronary computed tomography angiography (CCTA) images were triggered in diastole. Both IVUS and CCTA images were then used to reconstruct the pig-specific coronary artery geometries at T1 and T2. In detail, at each time point IVUS images were segmented into lumen contours with QCU-CMS software (Leiden, The Netherlands), and then aligned along the 3D centerline extracted from CCTA images using the MeVisLab software (MeVis Medical Solutions AG, Bremen, Germany). As previously reported[2], IVUS and CCTA images were matched using side branches as anatomical landmarks. The resulting geometries are displayed in Figure S1. Finally, the geometrical dimensions of each swine-specific coronary artery at time points T1 and T2 of the study are reported in Table S1 in terms of mean radius section and main vessel length.

Numerical Settings

Individual in vivo ComboWire Doppler velocity measurements were used to derive individualized boundary conditions according to the following strategy: (1) the inlet flow rate was estimated from the most proximal Doppler velocity measurement, and prescribed as inlet boundary condition in terms of time-dependent flat velocity profile; (2) the perfusion of side branches was quantified as the difference between Doppler velocity-based flow rate measurements taken upstream and downstream from each side branch and applied as outflow condition in terms of measured flow ratio. If velocity-based flow measurements were inaccurate or not available, a diameter-based scaling law[4] was applied to estimate the flow ratio at the outflow section[3,6]. No-slip condition was assumed at the arterial wall.

The computational fluid dynamics (CFD) code Fluent (ANSYS Inc., Canonsburg, PA, USA) was used on fluid domains discretized in ICEM CFD (ANSYS Inc., USA), by means of tetrahedrons and a 5-layers prismatic boundary layer. Blood was modelled as an incompressible (with density ρ equal to 1060 kg/m^3), non-Newtonian fluid using the Carreau model, belonging to the family of the generalized Newtonian fluids, whose dynamic viscosity is defined as:

$$\mu(\dot{\gamma}) = \mu_\infty + (\mu_0 - \mu_\infty)(1 + (\lambda\dot{\gamma})^2)^{\frac{n-1}{2}} \quad (1)$$

where, $\dot{\gamma}$ is the shear rate, and μ_∞ = 0.0035 kg m^{-1} s^{-1}, μ_0 = 0.25 kg m^{-1} s^{-1}, λ = 25 s, n = 0.25 [1]. Second order accuracy was prescribed to solve both the momentum equation and pressure with the COUPLED pressure-velocity coupling scheme. The backward Euler implicit scheme was adopted for time integration, with a fixed time increment defined as the measured swine-specific cardiac period divided by 100[3,5,6]. Convergence was achieved when the maximum mass and momentum residuals fell below 10^{-5}. All CFD setting (including mesh element size) were based on a sensitivity analysis[6], allowing only differences in terms of WSS lower than 1%.

Supplemental Results

The number and nature of instantaneous WSS fixed points at the coronary luminal surface was analysed and compared at time points T1 and T2. Their distribution, median and quartile range is presented in Figure S2. As expected, at T1 and T2 the number of WSS saddle points was higher than the number of WSS sinks and sources. Moreover, the occurrence of instantaneous WSS saddle points, sinks and sources was differently distributed at both time points T1 and T2, with saddle points distribution differing significantly from those of sinks (*p*=0.04 at T1 and *p*=0.0073 at T2, respectively; Figure S2) and sources (*p*=0.032 at T1 and *p*=0.0053 at T2; Figure S2). Conversely, no

significant differences emerged between sink and source occurrence (*p*=0.67 at T1 and *p*=0.93 at T2, respectively; Figure S2).

Figure S3 reports the luminal distribution of *TSVI* (quantifying the variation of WSS contraction/expansion action along the cardiac cycle) at T1 and T2, together with the distribution of measured WT at time points T1, T2 and T3 along the follow-up study, for all the investigated coronary models. By visual inspection it emerged that, in general, regions exhibiting high WT values markedly co-localized with luminal surface areas interested by a greater variation of WSS contraction/expansion action along the cardiac cycle (Figure S3). In detail, it resulted that (Figure S3): (1) at contemporaneous time points (i.e., *TSVI* at T1 *vs.* WT at T1; *TSVI* at T2 *vs.* WT at T2), luminal regions exposed to high *TSVI* values exhibited high WT values; (2) luminal surface areas exposure to high variation in WSS contraction/expansion action along the cardiac cycle at baseline corresponded to high WT values at T2 and T3; (3) the increase in *TSVI* from T1 to T2 corresponded to luminal regions of WT growth along the follow-up study (this is clearly evident at several locations highlighted by solid black circles in Figure S3); (4) the decrease in *TSVI* from T1 to T2 corresponded to luminal surface areas of WT regression from T2 to T3 (luminal regions highlighted by dashed black circles in Figure S3).

Tables S2 reports the odds ratios with associated confidence intervals quantifying the strength of the association between the luminal exposure to WSS-based quantities and high WT outcomes, as already displayed in Figure 5 of the main text.

Tables S3 reports the odds ratios with associated confidence intervals quantifying the strength of the association between the luminal exposure to WSS-based quantities and low WT outcomes, as already displayed in Figure 6 of the main text.

Supplemental Figures

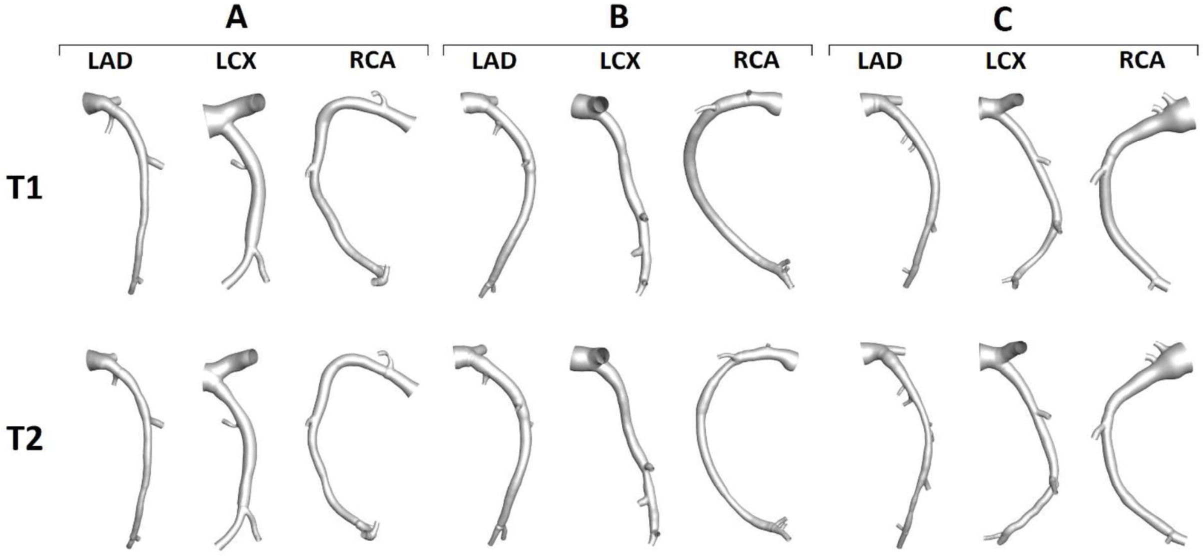

Figure S1. Geometry of the 9 swine coronary artery models at T1 and T2 of the follow-up time. Labels from A to C identify a single swine. For each swine, LAD, LCX and RCA geometries were reconstructed.

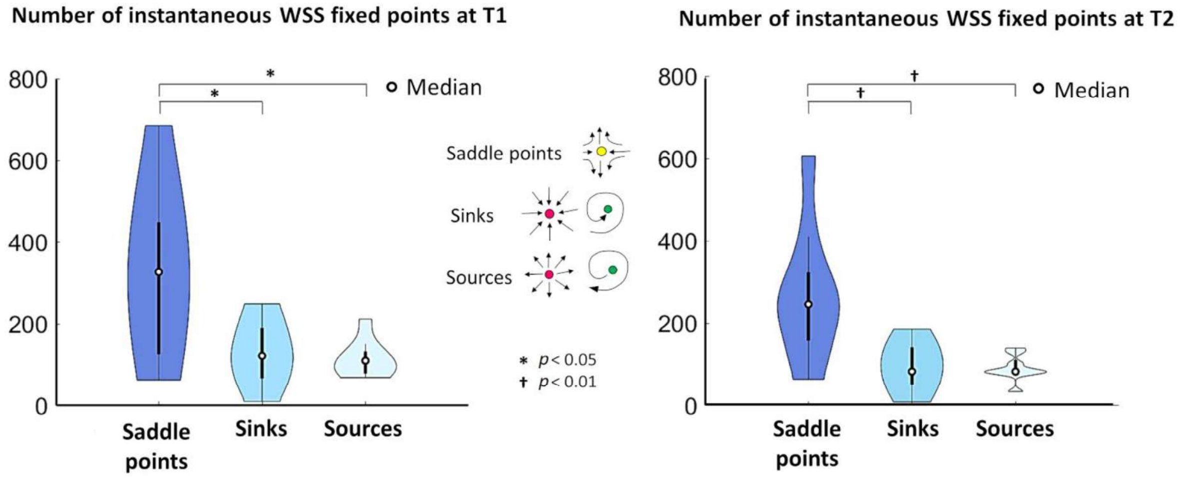

Figure S2. Violin plots of the prevalence of instantaneous WSS fixed points at the coronary luminal surface along the cardiac cycle. Distribution, median and quartile range are displayed for the occurrence of saddle points, sink and source at the coronary luminal surface along the cardiac cycle at time T1 and T2.

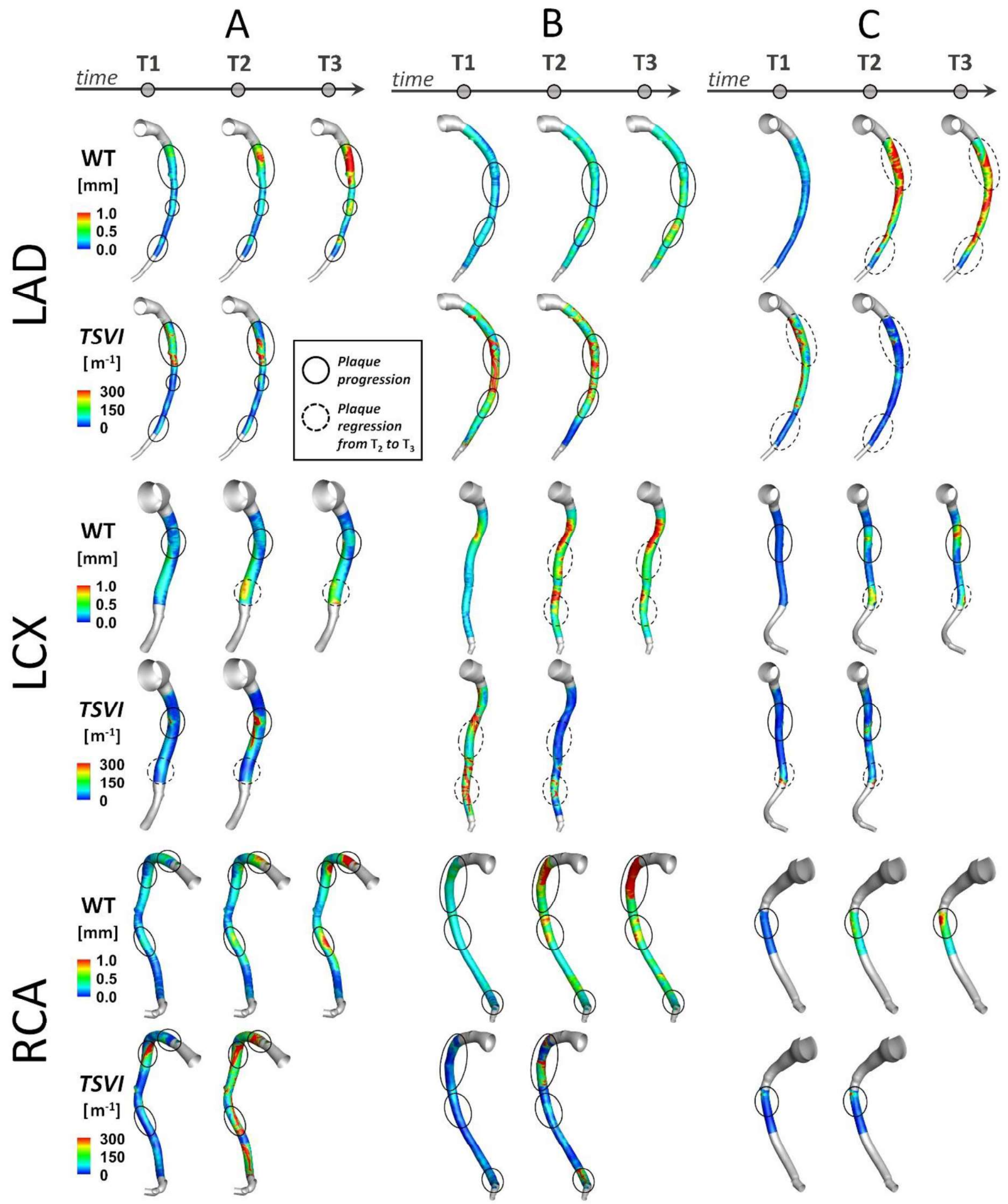

Figure S3. Luminal distributions of measured Wall Thickness (WT) and Topological Shear Variation Index (*TSVI*) at several time points along the follow-up study for all the investigate coronary artery models. The distributions of WT and *TSVI* are only shown along the IVUS segment of the main vessel. The side branches are not shown. The regions of interest identified along each vessel are emphasized by solid black circle if experiencing plaque progression over time or dashed black circle if experiencing plaque regression between T2 and T3.

Supplemental Tables

Table S1. Geometrical dimension of swine coronary models.

Swine model	T1		T2	
	mean radius [mm]	*vessel length [mm]*	*mean radius [mm]*	*vessel length [mm]*
A-LAD	3.62	8.17	3.42	8.19
A-LCX	3.66	4.65	3.31	4.72
A-RCA	4.20	11.18	3.80	11.20
B-LAD	4.31	7.70	4.24	8.79
B-LCX	3.75	5.82	3.05	6.31
B-RCA	4.27	11.01	3.72	11.31
C-LAD	3.46	7.32	3.17	7.56
C-LCX	2.71	6.60	2.50	6.85
C-RCA	3.60	5.25	3.42	5.81

Table S2. Odds ratio and associated confidence interval for each investigated association between hemodynamic events and high WT along the follow-up study. *p*-values are reported only if lower than 0.05.

WT at T3 > 66th			
**Hemodynamic Event**	**Odds Ratio**	**Confidence interval**	***p*-value***
TSVI at T1 > 80th	2.18	1.67-2.85	< 0.0001
TAWSS at T1 < 20th	4.25	3.23-5.59	< 0.0001
TSVI at T1 > 80th ∩ *TAWSS* at T1 < 20th	3.48	2.36-5.13	< 0.0001
TSVI at T2 > 80th	2.18	1.67-2.85	< 0.0001
TAWSS at T2 < 20th	1.88	1.44-2.46	< 0.0001
TSVI at T2 > 80th ∩ *TAWSS* at T2 < 20th	2.74	1.81-4.13	< 0.0001
**WT at T2 > 66th**			
**Hemodynamic Event**	**Odds Ratio**	**Confidence interval**	***p*-value***
TSVI at T1 > 80th	3.06	2.34-4.01	< 0.0001
TAWSS at T1 < 20th	4.08	3.10-5.37	< 0.0001
TSVI at T1 > 80th ∩ *TAWSS* at T1 < 20th	4.08	2.75-6.06	< 0.0001
TSVI at T2 > 80th	1.99	1.52-2.60	< 0.0001
TAWSS at T2 < 20th	1.34	1.02-1.75	< 0.05
TSVI at T2 > 80th ∩ *TAWSS* at T2 < 20th	1.94	1.29-2.09	<0.001
**WT at T1 > 66th**			
**Hemodynamic Event**	**Odds Ratio**	**Confidence interval**	***p*-value***
TSVI at T1 > 80th	2.63	2.01-3.44	< 0.0001
TAWSS at T1 < 20th	3.56	2.72-4.68	< 0.0001
TSVI at T1 > 80th ∩ *TAWSS* at T1 < 20th	4.62	3.09-6.90	< 0.0001

***** if $p < 0.05$

Table S3. Odds ratio and associated confidence interval for each investigated association between hemodynamic events and low WT along the follow-up study. *p*-values are reported only if lower than 0.05.

WT at T3 < 33rd			
**Hemodynamic Event**	**Odds Ratio**	**Confidence interval**	***p*-value***
TSVI at T1 < 20th	1.70	1.30-2.23	< 0.001
TAWSS at T1 > 80th	3.11	2.37-4.07	< 0.0001
TSVI at T1 < 20th ∩ *TAWSS* at T1 > 80th	3.59	2.28-5.66	< 0.0001
TSVI at T2 < 20th	0.96	0.73-1.28	
TAWSS at T2 > 80th	1.64	1.25-2.14	< 0.001
TSVI at T2 < 20th ∩ *TAWSS* at T2 > 80th	1.17	0.78-1.76	

WT at T2 < 33rd			
**Hemodynamic Event**	**Odds Ratio**	**Confidence interval**	***p*-value***
TSVI at T1 < 20th	2.21	1.69-2.90	< 0.0001
TAWSS at T1 > 80th	2.94	2.24-3.85	< 0.0001
TSVI at T1 < 20th ∩ *TAWSS* at T1 > 80th	4.01	2.53-6.36	< 0.0001
TSVI at T2 < 20th	1.11	0.84-1.46	
TAWSS at T2 > 80th	1.02	0.77-1.35	
TSVI at T2 < 20th ∩ *TAWSS* at T2 > 80th	0.71	0.46-1.10	

WT at T1 < 33rd			
**Hemodynamic Event**	**Odds Ratio**	**Confidence interval**	***p*-value***
TSVI at T1 < 20th	1.25	0.95-1.64	
TAWSS at T1 > 80th	2.57	1.97-3.37	< 0.0001
TSVI at T1 < 20th ∩ *TAWSS* at T1 > 80th	2.13	1.37-3.32	< 0.05

***** if $p < 0.05$

Supplemental References

1. Chiastra, C., D. Gallo, P. Tasso, F. Iannaccone, F. Migliavacca, J. J. Wentzel, and U. Morbiducci. Healthy and diseased coronary bifurcation geometries influence near-wall and intravascular flow: A computational exploration of the hemodynamic risk. *J. Biomech.* 58:79–88, 2017.
2. van der Giessen, A. G., M. Schaap, F. J. H. Gijsen, H. C. Groen, T. van Walsum, N. R. Mollet, J. Dijkstra, F. N. van de Vosse, W. J. Niessen, P. J. de Feyter, A. F. W. van der Steen, and J. J. Wentzel. 3D fusion of intravascular ultrasound and coronary computed tomography for in-vivo wall shear stress analysis: a feasibility study. *Int. J. Cardiovasc. Imaging* 26:781–796, 2010.
3. Hoogendoorn, A., A. M. Kok, E. M. J. Hartman, G. de Nisco, L. Casadonte, C. Chiastra, A. Coenen, S.-A. Korteland, K. Van der Heiden, F. J. H. Gijsen, D. J. Duncker, A. F. W. van der Steen, and J. J. Wentzel. Multidirectional wall shear stress promotes advanced coronary plaque development: comparing five shear stress metrics. *Cardiovasc. Res.* 116:1136–1146, 2020.
4. Huo, Y., and G. S. Kassab. Intraspecific scaling laws of vascular trees. *J. R. Soc. Interface* 9:190–200, 2012.
5. De Nisco, G., A. Hoogendoorn, C. Chiastra, D. Gallo, A. M. Kok, U. Morbiducci, and J. J. Wentzel. The impact of helical flow on coronary atherosclerotic plaque development. *Atherosclerosis* 300:39–46, 2020.
6. De Nisco, G., A. M. Kok, C. Chiastra, D. Gallo, A. Hoogendoorn, F. Migliavacca, J. J. Wentzel, and U. Morbiducci. The Atheroprotective Nature of Helical Flow in Coronary Arteries. *Ann. Biomed. Eng.* 47:425–438, 2019.
